# Only the anxious ones? Identifying characteristics of symptom checker app users: a cross-sectional survey

**DOI:** 10.1186/s12911-024-02430-5

**Published:** 2024-01-23

**Authors:** Anna-Jasmin Wetzel, Malte Klemmt, Regina Müller, Monika A. Rieger, Stefanie Joos, Roland Koch

**Affiliations:** 1 Institute for General Practice and Interprofessional Care, University Hospital Tübingen, Osianderstr 5, 72076 Tübingen, Germany; 2 Institute of Applied Social Sciences, Technical University of Applied Sciences, Würzburg-Schweinfurt, Tiepolostraße 6, 97070 Würzburg, Germany; 3 Institute for Philosophy, University of Bremen, Enrique-Schmidt-Str 7, 28359 Bremen, Germany; 4 Institute of Occupational Medicine, Social Medicine and Health Services Research, University Hospital Tübingen, Wilhelmstr 27, 72074 Tübingen, Germany

**Keywords:** Digital health, Mental health, eHealth, mHealth, Cyberchondria, Self-diagnosis, Symptom Checker, Patient safety

## Abstract

**Background:**

Symptom checker applications (SCAs) may help laypeople classify their symptoms and receive recommendations on medically appropriate actions. Further research is necessary to estimate the influence of user characteristics, attitudes and (e)health-related competencies.

**Objective:**

The objective of this study is to identify meaningful predictors for SCA use considering user characteristics.

**Methods:**

An explorative cross-sectional survey was conducted to investigate German citizens’ demographics, eHealth literacy, hypochondria, self-efficacy, and affinity for technology using German language–validated questionnaires. A total of 869 participants were eligible for inclusion in the study. As *n* = 67 SCA users were assessed and matched 1:1 with non-users, a sample of *n* = 134 participants were assessed in the main analysis. A four-step analysis was conducted involving explorative predictor selection, model comparisons, and parameter estimates for selected predictors, including sensitivity and post hoc analyses.

**Results:**

Hypochondria and self-efficacy were identified as meaningful predictors of SCA use. Hypochondria showed a consistent and significant effect across all analyses OR: 1.24–1.26 (95% CI: 1.1–1.4). Self-efficacy OR: 0.64–0.93 (95% CI: 0.3–1.4) showed inconsistent and nonsignificant results, leaving its role in SCA use unclear. Over half of the SCA users in our sample met the classification for hypochondria (cut-off on the WI of 5).

**Conclusions:**

Hypochondria has emerged as a significant predictor of SCA use with a consistently stable effect, yet according to the literature, individuals with this trait may be less likely to benefit from SCA despite their greater likelihood of using it. These users could be further unsettled by risk-averse triage and unlikely but serious diagnosis suggestions.

**Trial Registration:**

The study was registered in the German Clinical Trials Register (DRKS) DRKS00022465, DERR1-10.2196/34026.

**Supplementary Information:**

The online version contains supplementary material available at 10.1186/s12911-024-02430-5.

## Introduction

Symptom checker Apps (SCAs) are eHealth applications designed to support laypeople in assessing their symptoms and receiving recommendations on medically appropriate actions related to their health [1]. Users can input their health-related information into SCAs through a chatbot or search strings, and SCAs retrieve and categorize the input. Some SCAs are advertised as AI-based, and most generate healthcare-related information and recommendations for actions based on user input [2].

Although SCAs are already in use, their impact on healthcare systems remains poorly understood. Recent scoping reviews described ambiguous effects of SCAs [1, 3], indicating that they could both reduce or induce oversupply. The effectiveness of SCAs in delivering adequate and precise information and recommendations must be considered. Additionally, the possible impact of SCAs on healthcare systems depends on several factors, including the characteristics of SCAs and how SCAs are used. Finally, the impact of SCAs on users’ health-related behavior, such as seeking healthcare, must also be considered.

Recent studies have shown that the diagnostic accuracy and triage capabilities of SCAs are highly variable. A recent study reported a triage accuracy for primary conditions varying between 48.8% and 90.1% [1]. Additionally, a significant disparity in diagnostic accuracy between SCAs and emergency physicians has been reported. While SCAs correctly identified the primary diagnosis in only 30% of cases, emergency physicians achieved a much higher accuracy rate, successfully diagnosing 81% of cases [4]. In addition, another study found that medical laypeople still outperformed SCAs [5]. Consequently, SCAs currently struggle to reliably assist patients in navigating healthcare and addressing adequate medical recommendations.

Understanding the impact of SCAs on the healthcare system requires consideration of user demographics, such as (e)health literacy and attitudes toward technology [3, 6, 7]. Research indicates that SCA users are often female, well-educated, Caucasian, with health insurance and a regular healthcare provider [8, 9]. Recent studies showed that health literacy levels in Germany have declined over the years with the reported uncertainty being mainly related to online resources [10]. However, some users found SCAs useful for self-diagnosis and reported positive health effects [11], while others had problems giving and interpreting concrete information on symptom time patterns or severity [12]. Such difficulties may initiate unnecessary healthcare-seeking behavior, although the evidence remains inconclusive [13]. Additionally, increased eHealth literacy may lead to greater subjective trust in SCAs and the ability to critically evaluate their recommendations, but not necessarily to a change in actual trust-based behavior [14]. Lastly, user attitudes toward technology play a significant role, with “tech seekers” being more likely to use SCAs in the future compared to “tech rejectors” and “unsure acceptors” [15]. Concurrently with internet research, the usage of SCAs may also magnify preexisting user characteristics associated with unwarranted healthcare-seeking tendencies rather than operating independently [16]. As an example, SCA may worsen hypochondria, similarly to how internet research is already known to do among vulnerable patient groups [16].

There is a research gap concerning the influence of concepts such as hypochondria, self-efficacy, technology affinity, and health literacy on the use of SCAs. Therefore, the aim of this explorative study was to identify meaningful predictors for SCA use considering user characteristics.

## Methods

An explorative cross-sectional survey was conducted. The survey was available online or as a paper and pencil version. The STROBE (Strengthening the Reporting of Observational Studies in Epidemiology) checklist [17] was applied.

### Measurements

Due to the limited literature on SCAs, pilot interviews with SCA users and SCA experts were conducted to ensure a meaningful concept selection for the survey content. In addition, to identify potential characteristics of the user group, we drew on literature related to the use of health applications.

Thus, the following concepts were selected: eHealth literacy [18], hypochondria [19], self-efficacy [20], and affinity for technology [21]. Table 1 presents a comprehensive overview, detailing the reliability, validity, scale, and scoring of the evaluated scales (General Life Satisfaction Short Scale [22], German Version of the eHealth Literacy Scale [23], Whiteley Index [24, 25], General self-efficacy short scale [26], Ultra-Short Scale for Assessing Affinity for Technology Interaction [27]) used in this study.

Furthermore, the presence of chronic diseases, private screen time (as a potential indicator of smartphone use) were assessed. Sociodemographic variables such as age, gender, and school education were also assessed in this study.

**Table 1:** Overview, detailing the reliability, validity, scale, and scoring of the evaluated scales used in this study

Concept	Title of the Instument (Abbreviation)	Reliability	Validity	Number of Items	Scale	Score (Range)
Life satisfaction	General Life Satisfaction Short Scale (23) (L1)	retest = 0.82 (23)	evidence for construct validity (23)	1	10 point Likert	Sum Score (0–10)
eHealth literacy	German Version of the eHealth Literacy Scale (24) (G-eHeals)	Cronbach’s α = 0.83-0.88 (24)	construct validity was supported by correlation patterns with different scales and constructs (24)	8	5 point Likert	Sum Score (0–40)
Hypochondria	Whiteley Index (25) (WI)	Cronbach’s α = 0.76–0.8 (26)	high associations with health anxiety and illness behavior subscales (26)	14	binary („yes“ / „no“)	Sum Score (0–14)
Self-efficacy	General self-efficacy short scale (27) (ASKU-S)	McDonalds ω = 0.81 bis 0.87 (27)	Indications of content, factorial, convergent, discriminant and predictive validity (27)	3	5 point Likert	Mean Score (1–5)
Technic affinity	Ultra-Short Scale for Assessing Affinity for Technology Interaction (28) (ATI-S)	McDonalds ω=>0.8 (28)	sufficiently valid assessment (28)	4	6 point Likert	Mean Score (1–6)

### Recruitment

The survey was conducted from November 2020 to June 2021. The sample comprised different recruiting strands to reach a wide variety of participants and ensure a sufficient number of SCA users for the statistical analysis. In the first strand, *n* = 50.000 German citizens were contacted via mail to participate in the survey. The intended recipients were representatively selected by an external partner (T + R Dialog Marketing (Berlin, Germany) and Acxiom (Neu-Isenburg, Germany)). Further participants were recruited by mailing lists of the University of Tübingen and the University Hospital of Tübingen, social media and by cooperating GP practices. The second strand aimed to reach SCA users only; therefore, participants were only included if they had SCA experience. Targeted advertisements via social media, the social channels of the University Hospital of Tübingen, the homepage of a German newspaper and the social channels of federal health insurance were conducted to recruit further SCA users.

### Data exclusion

We assumed a missing completely at random mechanism (only single values were unplausible or missing, omitted by chance). Participants with missing data on the primary outcome were excluded (*n* = 2). Furthermore, physicians (*n* = 19) were excluded due to the assumption that their medical knowledge would have a significant influence on SCA usage.

### Statistical analysis

The primary outcome variable was whether participants had already used SCAs. Statistical analyses were conducted in different steps. The first step comprised variable selection using a least absolute shrinkage and selection operator (LASSO [28]) regularized logistic regression analysis considering nine predictors (as listed in Table 2). The second-step model comparison involved an intercept-only model, a full model and a model with the selected predictor using conventional logistic regression. In the third step, we utilized the identified predictors to derive parameter estimates and *p*-values. This process led to the determination of the main analysis parameters. A post-hoc analysis was conducted in the fourth step.

### Propensity score matching

Users and non-users were matched with propensity score matching [29, 30] on an initial set of potential confounders [31]. Confounder covariates included school education and age, as we assumed that we reached a younger and better-educated user population due to our targeted recruiting strategy via social media and university mailing lists. A nearest neighbor matching algorithm [29] was applied. Missing data on the predictors were imputed using a random forest approach [32] that enables the imputation of missing information in mixed-data (categorical and continuous). Out-of-bag errors were considered [32].

### Predictor selection using LASSO regularized logistic regression

The participants were divided into training (70%) and test (30%) data sets. The training data set was used to fit a model on the given data, and the test data set was used to evaluate the model [33]. A 0.632 bootstrap estimator [34] was applied as the resampling method for lambda selection. The sensitivity, specificity, Positive Predictive Value (PPV), Negative Predictive Value (NPV) and accuracy rate were calculated by fitting to the test data set. An overview of the predictors included can be found in Table 2.

### Model comparison of an intercept only, a full model and a model with the identified predictor

A conventional logistic regression was fit on the complete matched data set to derive odds ratios (ORs) and confidence intervals (CIs). To identify potential multicollinearity, we employed the Variance Inflation Factor (VIF), which assesses the variance of a coefficient within the full model in comparison to its variance when modeled independently [33]. A VIF value exceeding 5 were considered as indicative of significant collinearity [33]. The Akaike information criterion (AIC) of a full model, an intercept-only model and the model with the LASSO selected predictors was compared to assess model performance. The smaller the AIC, the better the performance of the model [33].

### Parameter estimators, CI and *p*-values of the models, including the selected predictors

Parameter estimators were derived from conventional logistic regression. Two sensitivity analyses were conducted to ensure the robustness of the ORs, CIs and *p* values considering different sample compositions. We applied a different matching algorithm (full optimal matching [29]) for the sensitivity analysis. Additionally, we used the whole sample without matching for sensitivity in the second analysis.

### Post hoc Analysis: categorization of the WI

Finally, a post hoc analysis was conducted considering a predictor identified in step 3. The variable was dichotomized to identify clinically relevant persons, and a Pearson’s χ^2^ test was conducted.

### Data Processing

Data processing and statistical analyses were conducted with R Version 4.1.1 [35, 36] and R Studio Version 1.4 [37].

## Results

A total of 869 participants (*n* = 116 paper-pencil, *n* = 753 online) completed the survey. As participants were matched 1:1 and 67 users finished the survey, the final analysis included *n* = 134 participants. The median age of the population was 31 (IQR 24–49), and 67% were female. The matched variables (age and school education) were well balanced between the user and non-user groups (Love Plot Supplemental Fig. 1).

Table 1 describes the matched sample stratified for SCA use, including all predictors used in the LASSO regression. Univariate analyses were conducted for all predictors. In addition to subjective rated health, hypochondria and self-efficacy showed a significant association with SCA use.

**Table 2:** Overview of the potential predictors stratified for SCA use and univariate analysis

Predictors	Non-user, *N* = 67^1^	User, *N* = 67^1^	*p*-value^2^
Gender			0.15
*Female*	49 (73%)	41 (61%)	
*Male*	18 (27%)	26 (39%)	
Chronic Disease	22 (33%)	31 (46%)	0.12
Subj. rated health	1.9 (0.8)	2.2 (0.7)	0.018
Life satisfaction (L1)	7.4 (1.6)	6.9 (1.5)	0.074
eHealth literacy (G-eHeals)	30.0 (5.6)	30.7 (5.4)	0.5
Hypochondria (WI)	2.6 (2.8)	4.8 (3.2)	< 0.001
Self efficacy (ASKU-S)	4.1 (0.6)	3.8 (0.7)	0.040
Technic affinity (ATI-S)	3.5 (1.2)	3.7 (1.1)	0.5
Private screen time			0.2
*0–30 min*	3 (4.5%)	2 (3.0%)	
*30–60 min*	14 (21%)	10 (15%)	
*1–2 h*	19 (28%)	19 (28%)	
*2–3 h*	15 (22%)	9 (13%)	
*3–4 h*	11 (16%)	13 (19%)	
*> 4 h*	5 (7.5%)	14 (21%)	

^1^n (%); Mean (Standard Deviation)

^2^chi-squared test with Rao & Scott’s second-order correction; Wilcoxon rank-sum test for complex survey samples

### Identification of meaningful predictors

**Fig. 1:**
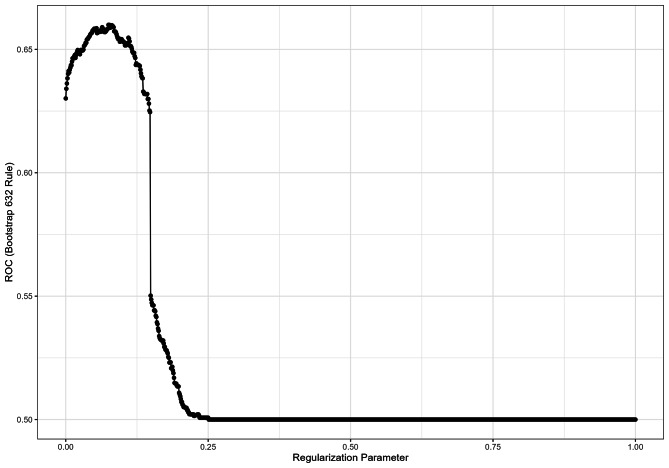
Bootstrapped ROC curve for λ

**Fig. 2:**
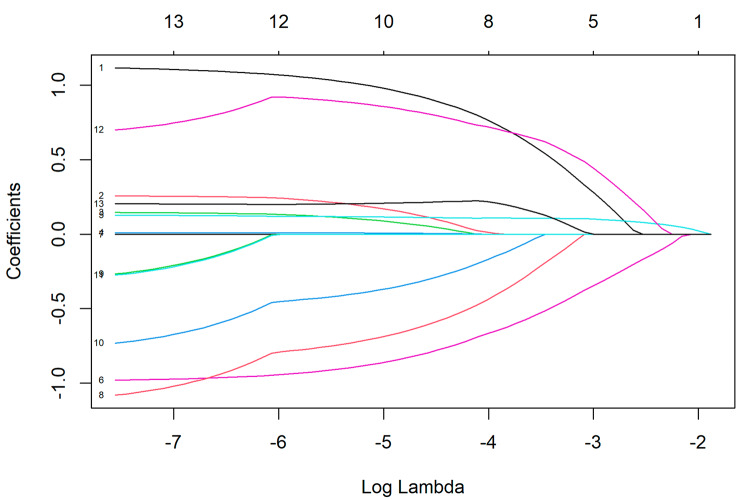
LASSO coefficient profiles against log (λ), Lambda = 0.112 when the error of the model is minimized, and 2 variables were selected

The training data set comprised 93 participants. The test data set comprised 41 participants. Nine variables were initially considered for predictor selection, as detailed in Table 2. The selection process, which involved a LASSO regularized logistic regression, identified two variables with nonzero coefficients: hypochondria (WI) and self-efficacy (ASKU). Consequently, these two variables were chosen as predictors in the conventional logistic regression model. The LASSO coefficient profiles against log (λ) are shown in Fig. 1, as is the bootstrapped ROC curve for the regularization parameter λ. Figure 2 shows the LASSO coefficient profiles against log (λ), Lambda = 0.112 when the error of the model is minimized, and 2 variables were selected. Sensitivity, specificity, Negative / Positive Predictive Values (NPV / PPV) and balanced accuracy can be found in Table 3.

**Table 3:** Model evaluation of the LASSO regression of the test data set

Sensitivity (%)	Specificity (%)	NPV (%)	PPV (%)	Balanced Accuracy
75.0	81.0	77.3	79.0	78.0

### Model comparisons

The AIC of the full model was 184.43, and the intercept-only model derived an AIC of 187.76. The logistic regression model based on the results of the LASSO variable selection (WI and ASKU-S) had the lowest AIC of 172.21 and therefore showed an improved performance compared to the full and intercept-only model. The VIF = 1.035 showed no considerable multicollinearity.

### Parameter estimators and predictor robustness

Table 4 shows the odds ratios, confidence intervals of the odds ratios, and *p* values of the logistic regression and its sensitivity analyses comprising the previously identified predictors, hypochondria and self-efficacy. The OR of the predictor hypochondria (WI) showed a similar value (1.24–1.26) for all three models and a significant *p* value (*P* <.001). The OR size corresponds to a small effect [38]. The ORs for self-efficacy, measured across the three models, were not statistically significant (*P* >.05). Additionally, the variation inflation factor for all models was low (VIF < 1.06), indicating no considerable multicollinearity.

**Table 4:** Results of the conducted logistic regression and the sensitivity analysis

	Resulting logistic regression			Sensitivity analysis 1			Sensitivity analysis 2		
Characteristic	OR^1^	95% CI^1^	*p*-value	OR^1^	95% CI^1^	*p*-value	OR^1^	95% CI^1^	*p*-value
Hypochondria (WI)	1.26	1.11, 1.46	< 0.001	1.24	1.14, 1.35	< 0.001	1.24	1.14, 1.35	< 0.001
Self efficacy (ASKU)	0.64	0.34, 1.15	0.14	0.93	0.63, 1.39	0.7	0.93	0.63, 1.39	0.7

^1^OR = Odds Ratio, CI = Confidence Interval

### Post hoc analysis

Pearson’s χ^2^ test revealed a significant difference between non-users and users among participants with clinically relevant levels of hypochondria on the WI.

Over half of the SCA users had a WI sum score higher than the cut-off of five, indicating clinically relevant hypochondria (Table 5).

**Table 5:** Post hoc analysis with categorized WI stratified for the user group

Hypochondria Scale (WI)	Non-user, *N* = 67^1^	User, *N* = 67^1^	*p*-value^2^
Continuous	2.0 (1.0, 3.5)	5.0 (2.0, 7.0)	< 0.001
Categorized with a cut-off > 5			< 0.001
*Hypochondria*	8 (12%)	35 (52%)	
*No hypochondria*	59 (88%)	32 (48%)	

^1^Median (IQR); n (%)

^2^Wilcoxon rank sum test; Pearson’s Chi-squared test

## Discussion

In this exploratory study, we identified WI-assessed hypochondria as a reliable predictor for SCA use. This predictor consistently affected all analyses, including the two sensitivity analyses. Furthermore, lower values of self-efficacy assessed with the ASKU-S were identified as a positive predictor for SCA use in the main analysis. The sensitivity analyses did not replicate the effect of this variable; thus, its role remains unclear due to the rather moderate sample size.

### Comparison with prior work

Hypochondria was identified as a predictor for SCA use and revealed a stable effect throughout our analyses. Over half of the SCA users had a WI sum score higher than the cut-off of five, indicating clinically relevant hypochondria (Table 4). This level of anxiety may affect a patient’s ability to adequately handle action recommendations and symptom classifications. Thus, these SCA users might be susceptible to the negative effects of SCA use. Hypochondria in the context of SCAs can be classified as cyberchondria, considering the working definition of Vismara [39]. A 2020 study discouraged self-diagnosis using SCAs among cyberchondriac patients and emphasized adjusting expectations accordingly when accessing health information online [40]. Another recent study revealed that some people with high WI (hypochondria) scores felt worse after online symptom checking, while others with low scores felt better [41]. Given this literature and our findings, it appears that patients with health anxiety are less likely to benefit from SCAs, despite being more inclined to use them. The transferability of results from online health-related use to SCAs is important to consider, as they suggest that prolonged use is associated with increased functional impairment and anxiety both before and after checking [41]. The impact of using SCAs on health-anxious patients remains unclear and warrants further investigation.

Furthermore, we examined self-efficacy as a meaningful predictor since a recent study indicated an association between self-efficacy and the adoption of SCA use [20]. The results of the predictor self-efficacy were ambiguous with differing effect sizes in our analyses. It is still uncertain how much self-efficacy contributes to determining the usage of SCAs.

Affinity for technology was another variable we considered since the literature indicated a potential association [15]. A study that examined SCA user profiles with a latent class analysis revealed that the latent class of “tech seekers” showed the highest odds of using SCA [15]. However, the results in our rather moderate sample do not suggest an association between affinity for technology and SCA use. Reasons for the discrepancy might be the different operationalization of the concepts (e.g., a scale rather than profiles) or the different study populations.

The broad use of SCAs can lead to individual- and systemic-level effects. SCAs could lead to a misuse of health care resources [4], such as users visiting emergency departments too early or too often. As a result, these users in nonurgent conditions put further strain on the health system by possibly increasing costs and taking resources from patients who need emergency care [3, 42]. To mitigate these risks, software developers should provide transparent information about the potential dangers of using SCAs. This information could be presented in the form of an instruction leaflet, available after downloading or using an SCA in a browser. The instruction should clearly state that using SCAs may increase health anxiety. The language used in the instruction should be concise and easy to understand so that users can absorb the information and take appropriate action.

The existing knowledge about SCAs should be used to improve SCA design in the best possible way and implement improvements to minimize the negative effects and strengthen the potential positive effects. Physicians should be trained to consider pre-informed patients and promote dialog. It is necessary to better understand the relationships between cyberchondria, hypochondria, and e-health literacy in the context of SCA use to derive recommendations for systemic interventions and plan targeted and helpful interventions.

### Strengths and limitations

In this study, we conducted an industry-independent investigation of SCA users. Furthermore, our research did not limit itself to a single SCA application; instead, we examined the usage patterns across various types of SCA applications, enhancing the generalizability of our findings. Additionally, by matching users and non-users based on age and education, we controlled for these variables, thereby strengthening the reliability of our analysis.

A limitation of this study is the cross-sectional design we employed. This approach restricts our ability to infer causal relationships between variables, as it only provides a snapshot in time, thereby limiting our understanding of the dynamics and directionality of the relationships observed. Additionally, the recruitment of this study lead to younger and better-educated individuals introduces a potential selection bias. Our study’s moderate sample size, while adequate for exploratory purposes, may not capture the full spectrum of SCA usage characteristics. Conducting this research on a larger scale would be beneficial to validate our findings and identify more nuanced predictors of SCA use. Moreover, our approach of double targeting SCA users to ensure a higher response rate might lead to response bias, potentially resulting in an overrepresentation of the views and behaviors of more engaged or interested users. In light of these limitations, future research should consider longitudinal studies involving more diverse and larger samples.

## Conclusions

Hypochondria emerged as a significant predictor of SCA use in our sample, with a consistently stable effect. Over half of the SCA users had clinically relevant hypochondria considering their values on the WI, which may impact their ability to handle SCAs effectively. According to the literature, persons with hypochondria are less likely to benefit from SCA. These users could be further unsettled by risk-averse triage and unlikely but serious diagnosis suggestions. Software developers should provide transparent information about the potential dangers of using SCAs, including that SCA use may increase health anxiety. Individuals with higher levels of health anxiety (hypochondria) might experience increased anxiety or functional impairment due to SCA use. Users should be cautious of over-relying on SCAs for health information and diagnosis. For healthcare professionals, training in addressing patient concerns arising from SCA use may be beneficial, particularly for managing individuals with high health anxiety. Further, the widespread use of SCAs may potentially lead to the misuse of healthcare resources, with nonurgent cases increasing the burden on emergency services.

### Electronic supplementary material

Below is the link to the electronic supplementary material.

Supplementary Material 1

Supplementary Material 2

Supplementary Material 3

## Data Availability

The datasets used and/or analysed during the current study are available from the corresponding author on reasonable request.

## Abbreviations

SCAs: Symptom Checker Apps
STROBE: Strengthening the Reporting of Observational Studies in Epidemiology
DRKS: German Clinical Trials Register
L1: General Life Satisfaction Short Scale
G-eHeals: German Version of the eHealth Literacy Scale
WI: Whiteley Index
ASKU-S: General self-efficacy short scale
ATI-S: Ultra-Short Scale for Assessing Affinity for Technology Interaction
LASSO: Least Absolute Shrinkage and Selection Operator
VIF: Variance Inflation Factor
PPV: Positive Predictive Value
NPV: Negative Predictive Value
OR: Odds Ratio
CI: Confidence Interval
