# Supplementary material for: Only the anxious ones? Identifying characteristics of symptom checker app users: a cross-sectional survey
Source: BMC Med Inform Decis Mak. 2024 Jan 23;24:21. doi: 10.1186/s12911-024-02430-5 (PMC10804572; doi:10.1186/s12911-024-02430-5)
Supplement: Supplementary file 1 — Supplementary Material 1 [file 12911_2024_2430_MOESM1_ESM.pdf]

EBERHARD KARLS UNIVERSITÄT TÜBINGEN

check.app

Institut für Allgemeinmedizin und Interprofessionelle Versorgung
Universitätsklinikum Tübingen

Questionnaire of the research project Check.App

Instructions

Only one box should be selected, if not stated otherwise. → ☒ yes ☒ no

To fix a made mistake, please fill out the wrongly chosen box completely and tick the right box. → ■ yes ☒ no

<u>For further corrections,</u> the second wrongly chosen box should be filled out completely and the new tick should be placed above the correct box. → x ■ yes ■ no

1. **Do you have access to the internet at home?** ☐ no ☐ yes

2. **What kind of devices do you use to access the internet?**
   It is possible to choose more than one answer
   ☐ computer ☐ tablet ☐ smartphone ☐ laptop
   ☐ none ☐ others: ______________________________

3. **Do you use applications or portable devices (e.g., smart watch) which collect data of your body measurements and functions and / or evaluate it?** ☐ no
   ☐ yes, these are…? ______________________________

4. **Which functions do you track with applications or portable devices?**
   It is possible to choose more than one answer
   ☐ sleep ☐ weight ☐ blood pressure ☐ steps
   ☐ active minutes ☐ menstrual cycle ☐ fluid intake ☐ nutrition ☐ heart rate
   ☐ I do not track any body measurements or functions

5. **Please estimate the amount of time per day which you spend using your smartphone or tablet in privat.**
   ☐ 0-30 minutes ☐ 30-60 minutes ☐ 1-2 hours
   ☐ 2-3 hours ☐ 3-4 hours ☐ 4 hours and more
   ☐ I do not have a smartphone or tablet

6. **What is your general state of health?**
   It is very good, good, moderate, bad or very bad?

very good	good	moderate	bad	very bad
☐	☐	☐	☐	☐

7. Assuming you're having health issues, from where do you retrieve information mostly?
It is possible to choose more than one answer

☐ friends / acquaintances ☐ books / magazines ☐ internet (e.g., search engine Google)

☐ symptom checker ☐ GP / doctors

Do you have a GP (general practitioner)? ☐ yes ☐ no

a. If yes, how often do you visit your GP?

☐ more often ☐once a month ☐ once every 3 months

☐ once in half a year ☐once a year ☐ less often

ZAPA

The following statements concern your satisfaction with your GP in general (which means, not only concerning your last visit).

Do you trust in your GP?	yes, I do trust my GP a lot ☐	yes, I do rather trust my GP a lot ☐	I do rather trust my GP less ☐	no, I don't trust my GP ☐
How satisfied are you with your GP in general, regarding the quality and quantity of information, which has been provided?	very satisfied ☐	rather satisfied ☐	rather unsatisfied ☐	very unsatisfied ☐
How satisfied are you with your GP in general, regarding your involvement in medical decisions?	very satisfied ☐	rather satisfied ☐	rather unsatisfied ☐	very unsatisfied ☐
How do you assess the quality of treatment of your GP in general?	very high ☐	rather high ☐	rather low ☐	very low ☐

L-1

	not satisfied at all									completely satisfied
The following is concerning your general satisfaction. How satisfied are you currently with all aspects of your life?	☐ 1	☐ 2	☐ 3	☐ 4	☐ 5	☐ 6	☐ 7	☐ 8	☐ 9	☐ 10

8. Have you ever heard of so-called "symptom checkers" that can help assess existing symptoms?

☐ no, I have never heard of symptom checkers before.

☐ yes, I have heard of it, but I never used it.

☐ yes, I have already used a symptom checker.

a. If yes, which symptom checkers have you heard of?
It is possible to choose more than one answer

☐ Ada ☐ Babylon ☐ WebMD ☐ Symptomate

☐ Isabel Symptom Checker ☐ Diagnose Medizin App ☐ Net Doktor Symptom Checker

☐ others: ______________________________

b. If you have *not yet used a symptom checker* or *not yet heard of it*:

Would you be interested in trying out using Symptom Checker? ☐ yes

☐ no, because:______________________________

9. Have you ever tried using one of the following symptom checkers?

☐ yes ☐ no (please proceed to the next page)

a. Which symptom checker have you tried?
It is possible to choose more than one answer

☐ Ada ☐ Babylon ☐ WebMD ☐ Symptomate

☐ Isabel Symptom Checker ☐ Diagnose Medizin App ☐ Net Doktor Symptom Checker

☐ others:______________________________

b. How satisfied have you been with the use of symptom checkers?

very satisfied	rather satisfied	neither nor	rather unsatisfied	very unsatisfied
☐	☐	☐	☐	☐

c. What do you like about symptom checkers:

____

____

d. **What don't you like about symptom checkers?**

____

____

G-eHeals

The following statements can be more or less applicable to you. Please indicate for each of these statements, how much you do agree with it.

	strongly disagree	disagree	neutral	agree	strongly agree
I know how to use the internet to find useful health information.	☐	☐	☐	☐	☐
I know how to use the internet to find answers to all my health-related questions.	☐	☐	☐	☐	☐
I know which sources for health information on the internet are available.	☐	☐	☐	☐	☐
I know where on the internet to look for useful health information.	☐	☐	☐	☐	☐
I know how to use information provided on the internet, so that they can help me.	☐	☐	☐	☐	☐
I'm able to evaluate information critically, which I find on the internet.	☐	☐	☐	☐	☐
I'm able to distinguish reliable and questionable information provided on the internet.	☐	☐	☐	☐	☐
If I make health-related decisions based on information provided on the internet, I feel secure.	☐	☐	☐	☐	☐

ATI-S

The following concerns your interaction with technical systems. The term ‚technical systems' includes applications and other application softwares, as well as whole digital devices (e.g., smartphone, computer, television, car navigation).

Please provide the level of **agreement** to the following statements.

	not true at all	usually not true	rarely true	often true	usually true	definitely true
I like to deal closely with technical systems.	☐	☐	☐	☐	☐	☐
I like to test functions of new techical systems.	☐	☐	☐	☐	☐	☐
It's enough for me, if a technical system works, I don't care how and why.	☐	☐	☐	☐	☐	☐
It's enough for me, to know the main functions of a technical system.	☐	☐	☐	☐	☐	☐

ASKU

The following statements can be more or less applicable to you. Please indicate for each of these statements, how much they apply with you personally.

	completely disagree	mostly disagree	slightly agree	mostly agree	completely agree
In difficult situations, I can rely on my abilities.	☐ 1	☐ 2	☐ 3	☐ 4	☐ 5
Most problems I can deal with by myself using my own strength.	☐ 1	☐ 2	☐ 3	☐ 4	☐ 5
Even exhausting and complicated tasks I can usually solve well by myself.	☐ 1	☐ 2	☐ 3	☐ 4	☐ 5

**WI-D**	yes	no
Please answer the following questions, by selecting either "yes" or "no".		
Are you often worried, that you might have a serious illness?	☐	☐
Are you being bothered by a variety of pains?	☐	☐
Are you often aware of the various processes, that occur inside your body?	☐	☐
Are you worried about your health?	☐	☐
Are you often experiencing symptoms of a very serious illness?	☐	☐
If an illness is brought to your attention (by the radio, television, newspaper or an acquaintance), are you worried then, that you could also get this illness?	☐	☐
If you feel sick and someone tells you, that you are looking better already – does it bother you?	☐	☐
Do you think that you are being bothered by a variety of different symptoms?	☐	☐
Is it difficult for you, once not to think about you, but instead about all other possible things?	☐	☐
Can you only hardly believe your doctor, when he/she tells you that there is no reason to worry about?	☐	☐
Do you feel that people do not take your illness seriously?	☐	☐
Do you think that you are worried about your health, more than other people?	☐	☐
Do you think that there is something seriously not in order with your body?	☐	☐
Are you afraid of getting sick?	☐	☐

10. Year of birth: (e.g. 1990) ☐

11. Gender:

☐ female ☐ male ☐ diverse

12. Do you live in an urban or rural area?

☐ city (more than 100.000 inhabitants) ☐ suburban area

☐ medium or small town (5.000-100.000 inhabitants) ☐ rural village

☐ single farmstead or house in the country

13. Are you and both of your parents born in Germany?

This question only serves to check the representativeness.

☐ no ☐ yes

14. What is the highest level of education you have completed?

☐ no school completed ☐ polytechnic secondary school certificate

☐ school-leaving qualification ☐ high school certificate

☐ secondary school certificat

15. What is your highest level of education?

☐ none ☐ apprenticeship

☐ "subject-specific higher education entrance qualification ☐ college degree

☐ bachelor's degree / master's degree / doctoral degree (university)

16. You are currently...?

☐ working ☐ job-seeking ☐ student ☐ pupil

☐ apprentice ☐ homemaker ☐ retired

17. What is your profession?

____

18. Are you in your current job…

☐ …self-employed (own business, workplace, ...)

☐ …employed (worker, employee or official)

19. Are you working in the medical field?

e.g. doctor, medical assistant, physio therapist or nurse

☐ no

☐ yes, as:____

20. Do you suffer from a chronic disease?

Chronic diseases are long-lasting illnesses, which can not be cured completely and result in a permanent or recurring use of healthcare services (e.g. physio therapy or occupational therapy)

☐ no

☐ yes, I have (multiple answers allowed):

____

____

☐ N/A (not applicable)

Thank you very much for your participation!
