# Supplementary material for: Only the anxious ones? Identifying characteristics of symptom checker app users: a cross-sectional survey
Source: BMC Med Inform Decis Mak. 2024 Jan 23;24:21. doi: 10.1186/s12911-024-02430-5 (PMC10804572; doi:10.1186/s12911-024-02430-5)
Supplement: Supplementary file 2 — Supplementary Material 2 [file 12911_2024_2430_MOESM2_ESM.docx]

**Supplementary Information**

The tidyverse (1) packages were used to clean and explore data. The *ggplot2* (2) package was used to create figures. Tables were created with the packages *srvyr* (3) and *gtsummary* (4)*.* The *glmnet* package was used to conduct LASSO regularized logistic regression. The R package *missForest (5)* was used to impute missings. The *MatchIt (6)*  package were used to realize matching.

1. Wickham H, Averick M, Bryan J, Chang W, McGowan LDA, François R, et al. Welcome to the tidyverse. J Open Source Softw. 2019;4(43):1686.

2. Wickham H, Chang W, Wickham MH. Package ‘ggplot2’. Create elegant data visualisations using the grammar of graphics. Version. 2016;2(1):1-189.

3. Ellis GF, Lumley T, Zółtak T, Schneider B, Krivitsky PN, Ellis MGF. Package ‘srvyr’ 2018 [Available from: <https://cran.rproject.org/web/packages/srvyr/srvyr.pdf>.

4. Sjoberg DD, Whiting K, Curry M, Lavery JA, Larmarange J. Reproducible summary tables with the gtsummary package. R J. 2021;13(1):570-80.

5. Stekhoven DJ, Buhlmann P. MissForest--non-parametric missing value imputation for mixed-type data. Bioinformatics. 2012;28(1):112-8.

6. Ho D, Imai K, King G, Stuart E, Whitworth A. Package ‘MatchIt’. Version[Google Scholar]. 2018.
