# Supplementary figures and images for: Only the anxious ones? Identifying characteristics of symptom checker app users: a cross-sectional survey
Source: BMC Med Inform Decis Mak. 2024 Jan 23;24:21. doi: 10.1186/s12911-024-02430-5 (PMC10804572; doi:10.1186/s12911-024-02430-5)

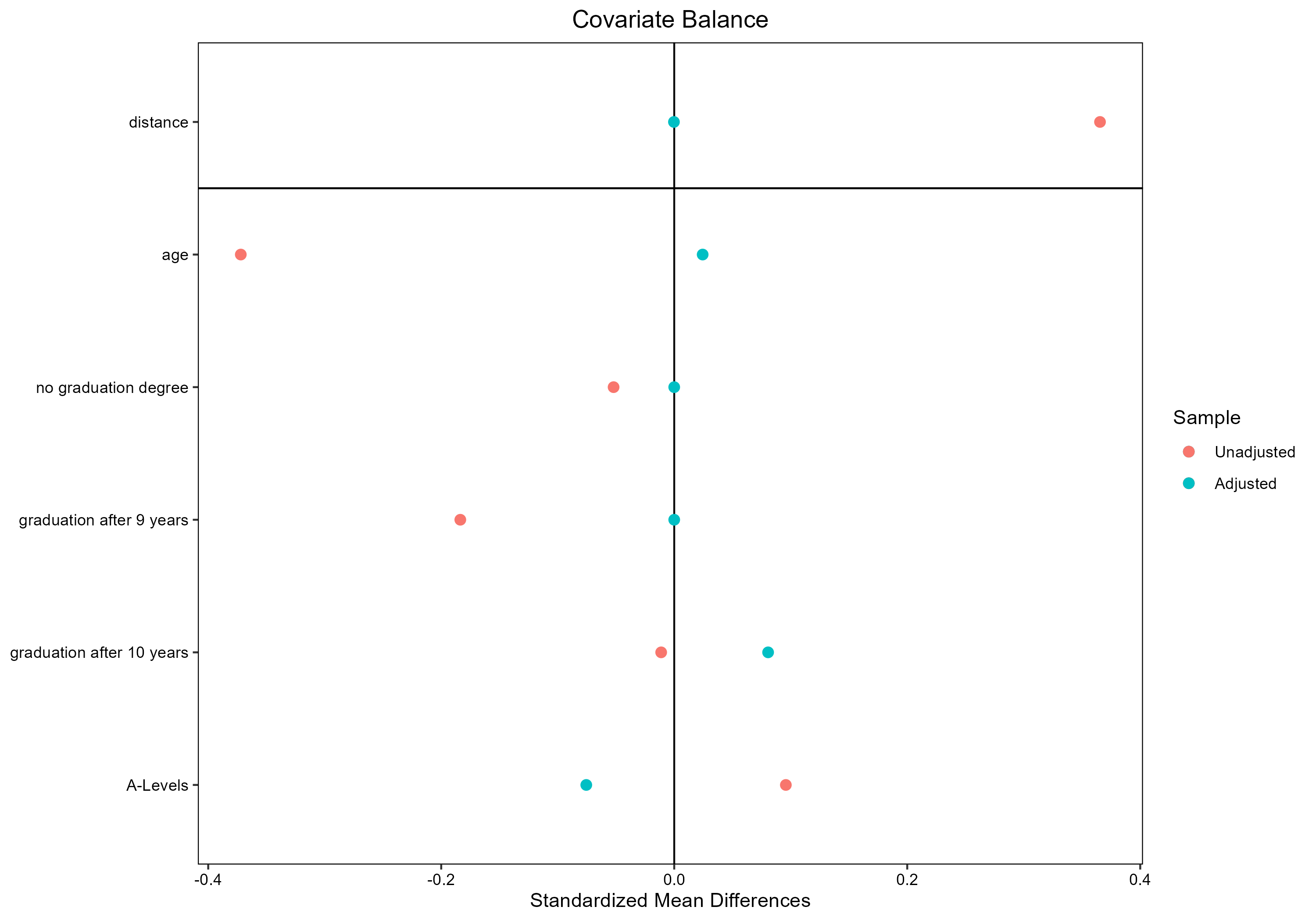

Supplement: Supplementary file 3 — Supplementary Material 3 [file 12911_2024_2430_MOESM3_ESM.tiff]
